# Effectiveness of face masks used to protect Beijing residents against particulate air pollution

**DOI:** 10.1136/oemed-2017-104765

**Published:** 2018-04-09

**Authors:** John W Cherrie, Andrew Apsley, Hilary Cowie, Susanne Steinle, William Mueller, Chun Lin, Claire J Horwell, Anne Sleeuwenhoek, Miranda Loh

**Affiliations:** 1 Institute of Occupational Medicine, Centre for Human Exposure Science, Edinburgh, UK; 2 Institute of Biological Chemistry, Biophysics and Bioengineering, Heriot-Watt University, Edinburgh, UK; 3 School of Chemistry, University of Edinburgh, Edinburgh, UK; 4 Department of Earth Sciences, Institute of Hazard, Risk and Resilience, Durham University, Durham, UK

**Keywords:** exposure assessment, ppe, air pollution, diesel fumes, pm10-pm2.5-ultrafine

## Abstract

**Objectives:**

Many residents in Beijing use disposable face masks in an attempt to protect their health from high particulate matter (PM) concentrations. Retail masks may be certified to local or international standards, but their real-life performance may not confer the exposure reduction potential that is marketed. This study aimed to evaluate the effectiveness of a range of face masks that are commercially available in China.

**Methods:**

Nine masks claiming protection against fine PM (PM_2.5_) were purchased from consumer outlets in Beijing. The masks’ filtration efficiency was tested by drawing airborne diesel exhaust through a section of the material and measuring the PM_2.5_ and black carbon (BC) concentrations upstream and downstream of the filtering medium. Four masks were selected for testing on volunteers. Volunteers were exposed to diesel exhaust inside an experimental chamber while performing sedentary tasks and active tasks. BC concentrations were continuously monitored inside and outside the mask.

**Results:**

The mean per cent penetration for each mask material ranged from 0.26% to 29%, depending on the flow rate and mask material. In the volunteer tests, the average total inward leakage (TIL) of BC ranged from 3% to 68% in the sedentary tests and from 7% to 66% in the active tests. Only one mask type tested showed an average TIL of less than 10%, under both test conditions.

**Conclusions:**

Many commercially available face masks may not provide adequate protection, primarily due to poor facial fit. Our results indicate that further attention should be given to mask design and providing evidence-based guidance to consumers.

**Key messages**

**What is already known about this subject?**

- Both the filtration efficiency and edge-seal leakage of a face mask or respirator are important in determining the exposure reduction a mask will confer on the wearer.
- Mask wearing is associated with positive impacts such as short-term reductions in blood pressure and increases in heart rate variability, but the exposure reduction associated with these impacts is not known.

**What are the new findings?**

- Filtration efficiency of a face mask does not necessarily translate into consistent exposure reduction for individuals.
- Some masks marketed as highly efficient at reducing particulate exposure do not achieve the claimed exposure reduction when worn by individuals.
- Even the best performing masks did not always reduce exposure consistently across a range of activities.

**How might this impact on policy or clinical practice in the foreseeable future?**

- This study indicates that rigorous and standardised testing, including volunteer trials, should be conducted to ensure the efficacy of all face masks available for consumer use against air pollution.
- Furthermore, consumers and regulators should be better informed about the exposure impacts of different mask types and how to ensure that masks fits appropriately.
- Researchers interested in investigating the health impacts of face masks should include measurements of exposure reduction on study participants to more accurately evaluate the impact of mask wearing on air pollution exposure.

## Background

In China, air pollution causes around 1.6 million premature deaths each year and the loss of around 31 million disability-adjusted life years.^1^ Annual average particulate matter of aerodynamic diameter 2.5 micrometres (PM_2.5_) concentrations in Chinese megacities well exceed the WHO’s guideline of 10 µg/m^3^, and corresponding black carbon (BC) concentrations are around 5 µg/m^3^.^2^ Beijing PM_2.5_ concentrations often reach unhealthy levels during the winter and near busy streets.^3^

Chinese residents now have access to air quality information and can take measures to protect themselves in the short term. Studies in China have found that wearing face masks can have positive impacts on short-term health outcomes such as blood pressure and heart rate variability,^4–6^ although it is unclear what level of exposure reduction was associated with these impacts, given that only ambient particulate concentrations were measured.

In occupational health, the term ‘respirator’ includes a range of personal respiratory protection products, including disposable filtering facepiece (FFP) respirators, sometimes referred to as ‘dust masks’ or ‘face masks’, particularly in lay parlance. In this manuscript, we use the latter term (or abbreviate to ‘mask’), as we are evaluating these devices for use in non-occupational air pollution settings. Respirators remove particles from the inhaled airstream by filtration, through gravitational settling, inertial impaction, interception, diffusion and electrostatic deposition.^7^ Filtration efficiency depends on particle size, charge, concentration and flow rate through the filter material. In practice, the contaminant may bypass the filter, by passing through small gaps between the edge of the respirator and the face, so-called ‘edge-seal leakage’. The extent of leakage depends on factors such as the size and shape of the face, facial hair, the respirator design and the way that it is worn.

Disposable particulate respirators sold for use in workplaces are generally tested to ensure compliance with appropriate international or national standards (online supplementary table S1), but face masks sold for public protection against air pollution may not need to comply with any standards, and so may not be suitable. In November 2016, the Chinese National Institute of Standardization issued a new guideline to recommend testing of protection levels for ‘daily protective masks’ (online supplementary table S1). This guideline (GB/T 32 610–2016) categorises masks based on their filtration efficiency (online supplementary table S1). Unlike GB2626-2006 (the Chinese standard for occupational masks) and other regulatory standards, it is not compulsory that ‘daily protective masks’ fulfil these testing guidelines. The tests in this study were carried out before the introduction of this guideline.

10.1136/oemed-2017-104765.supp1 Supplementary file 1

There is limited information about the performance of face masks against diesel exhaust particulate, which may play an important role in the health impacts of ambient particulate matter (PM).^8 9^ Studies often test the filtration efficiency of a section of the mask filter material in a sample holder or on mannequin heads, usually sealing the mask to the mannequin’s face. One study investigated the filtration efficiency of mask filter material complying with the N95 criteria (filtering ≥95% particulate, online supplementary table S1),^10^ finding that between <0.5% and 4.3% of the diesel aerosol, measured as elemental carbon, passed through the filters. Langrish *et al*^4^ demonstrated that several mask materials allowed <5% of a diesel exhaust challenge through the material. A study of the filtration efficiency of masks mounted on a mannequin head with mask sealed to the head found penetration of diesel aerosol mass between 11% and 15% for FFP2 masks and 13%–24% for FFP3 masks.^11^ These studies, however, did not account for inward leakage around the seal between the mask and face on humans.

Previous work on face masks used by the public has indicated poor performance, with masks often being poorly designed and ill-fitting.^12–14^ The Shanghai Municipal Bureau of Quality and Technical Supervision and China Consumers Association tested the filtration efficiency of several commercially available face masks.^15 16^ The methods are not described in the reports, but few of the masks passed the requirements. We have found no direct measurements of the effectiveness of masks against diesel exhaust particulate or ambient particulate pollution when worn by people. Masks mounted on mannequins provide only partially realistic tests. Therefore, the aim of this study was to test the filtration efficiency of a range of masks sold to consumers in Beijing and, for the best performing masks, to assess mask effectiveness in reducing exposure to diesel exhaust particulates when worn by volunteers.

## Methods

**Figure 1:**
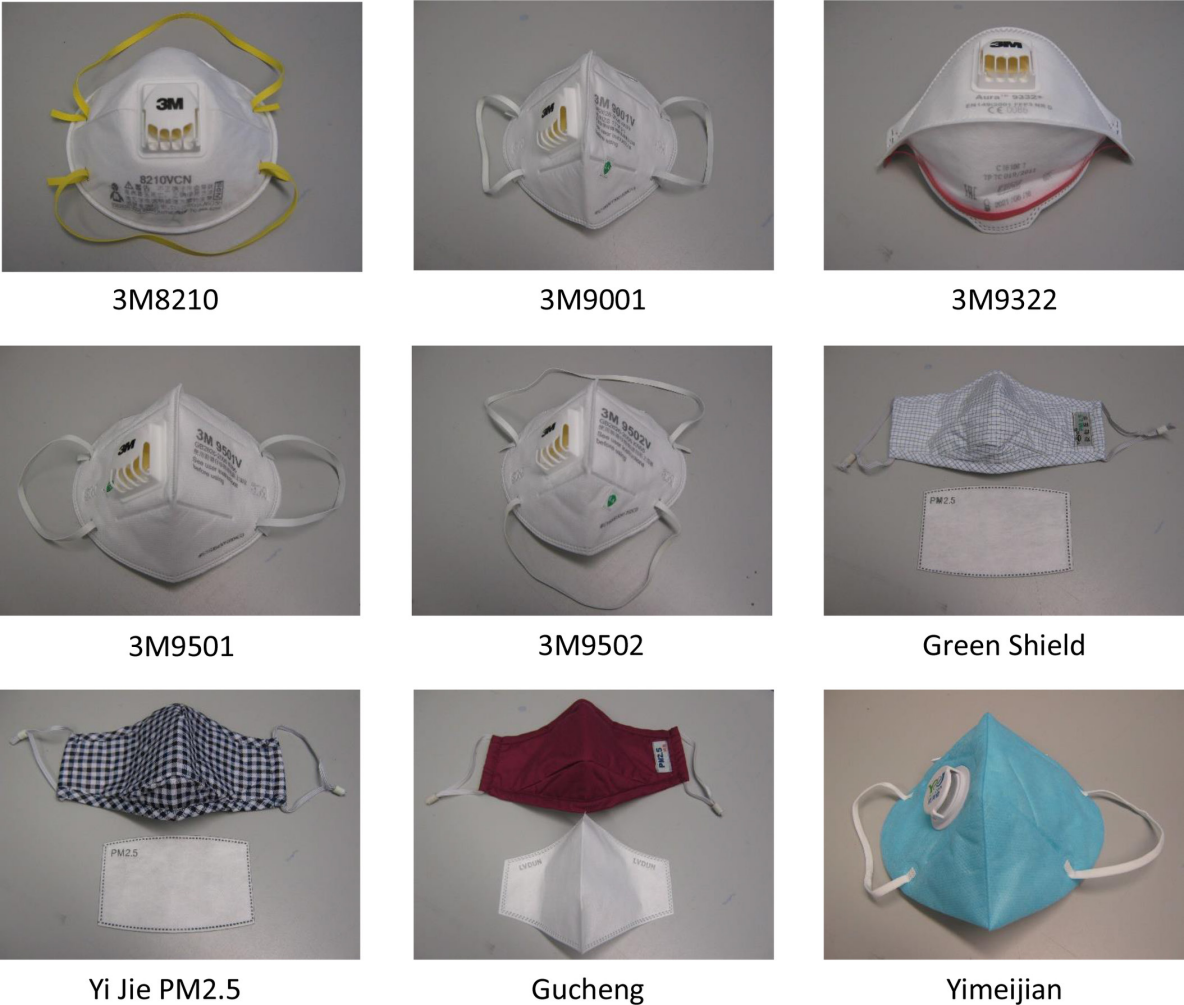
Pictures of the masks examined in this study.

Nine masks were purchased in China (figure 1, online supplementary table S2), online, in a local pharmacy or convenience store. Tests were conducted in a chamber in Edinburgh, UK. Prior to the mask testing, the diesel particulate was characterised using a DustTrak DRX (TSI, Minnesota, USA) and Fast Mobility Particle Sizer (FMPS 3091, TSI).

### Mask material penetration

The surface area of each mask was estimated using image editing software (Paint.net V.4.013, dotPDN LLC, 2016) from a scanned image. A circular area was cut from each mask and fitted to a sample holder (15.9 cm^2^).

Masks were tested in a chamber (online supplementary figure S1) for 30 min under flow rates equivalent to 40 L/min and 80 L/min through the whole mask but scaled to the area of the mask in the sampler. These rates were chosen to reflect high intensity breathing rates, which would be expected to draw more particles through the filter material and therefore mimic a worst case scenario (eg, cycling or running through traffic). The US Environmental Protection Agency (EPA)’s Exposure Factors Handbook, suggests the mean high activity breathing rate for males and females aged 21–<31 years old is 50 L/min (95th percentile 76 L/min).^17 18^

The testing order of the masks was randomised. The target challenge BC concentration was 50 µg/m^3^. A MicroAeth AE51 (Aethlabs, San Francisco, USA) measured BC, and a Sidepak AM510 (TSI) measured PM_2.5_ (online supplementary information). The Sidepak was not calibrated for diesel exhaust, because we were examining the relative change in filtration, rather than absolute concentrations.

### Volunteer tests

Four masks for the volunteer tests were selected based on performance in the material penetration tests and mask design, including how the masks would seal against the face and method of attaching the mask to the head.

Volunteers were recruited via word of mouth, social media and a local marketplace website. Volunteers were screened to ensure they had no underlying health problems. The original design was to have eight volunteers (four male, four female), each wearing all four masks, with two tests per mask (one standing and one sitting). However, the first two female volunteers dropped out after testing two masks, and two other female volunteers were recruited to test the remaining two masks. The total number of volunteers, therefore, was 10 (4 male, 6 females) (see online supplementary table S3).

Four facial dimensions were measured using anthropometric callipers: lip length, distance from chin to nasal root (menton–sellion length), facial width (bizygomatic width) and tragion (ear) to the tip of the nose. One individual measured all of the volunteers to reduce any variability in the measurement method. A Latin square design was employed to randomise the order in which the masks were tested by the volunteers. Additional information on the mask and sampling equipment donning are available in online supplementary information.^19^

The tests were conducted in an exposure chamber supplied with air from a mixing chamber connected to a small diesel engine (online supplementary figure S2) with the target average BC concentration of 50 µg/m^3^.

Volunteers performed two simulation tests, designed to reflect everyday activities: (1) sitting (sedentary) and (2) standing and walking on the spot (active). All simulations had subtasks: breathing normally (5 min), deep breathing (2 min), moving the head up and down (2 min), moving the head side to side (2 min), talking (2 min), bending over (2 min) and breathing normally (2 min). BC was measured simultaneously inside the mask (penetration concentration) and in the exposure chamber (challenge concentration).

### Data analysis

The per cent penetration for each mask’s filter material was calculated by dividing the penetration concentration by the challenge concentration at each time point, for BC and PM_2.5_. Agreement between the penetration percentages from the MicroAeth and Sidepak readings were assessed using a Spearman correlation. For the simulation study, the total inward leakage (TIL) percentage of BC was calculated by dividing the concentration measured inside each mask by the concentration in the exposure chamber for each time point.

The unadjusted mean, median and interquartile range (IQR) of TILs were generated for each mask. Autocorrelation for time-series data, where values are influenced by previous time points, was assessed with the Durbin-Watson statistic.^20^ A Cochrane-Orcutt first-order autoregression model was developed to adjust TILs by the following covariates: mask type, time, gender, face size and activity. Covariates were included in the model using a likelihood ratio test significant at P<0.05. Goodness of fit of a log-linear model was compared using the Bayesian information criterion. For categorical variables, the level contributing the smallest incremental increase in TIL was used as the reference. The activities undertaken during the tests were grouped if volunteers were (1) sitting or standing/walking, (2) moving their head, (3) remaining stationary, (4) talking, (5) breathing deeply or (6) bending. Volunteers were classified as either small/medium or large face sizes by National Institute for Occupational Safety and Health (NIOSH) guidelines based on face length and width^21^ and through the assessment of lip length and face depth. Data analysis was performed using the ‘prais’ command in Stata V.13.1.μ

## Results

During the diesel characterisation period, the chamber air PM_2.5_ consisted of >90% PM_1_. The nanoparticle size distribution in the chamber indicated that particles were primarily below 0.3 µm, with the mode below 0.1 µm.

### Mask material penetration

**Figure 2:**
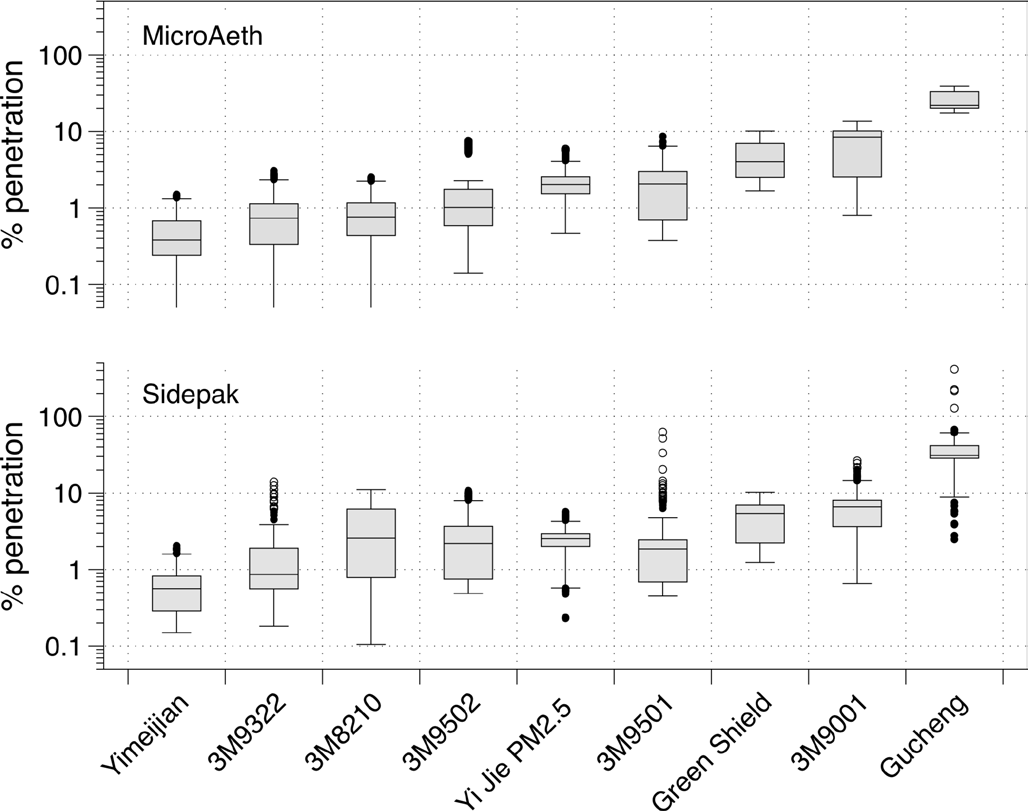
Boxplots of the per cent penetration of each mask assessed for black carbon (MicroAeth) and PM_2.5_ (Sidepak) monitors. Boxes indicate the 25th–75th percentiles (the IQR), and the lines inside the boxes indicate the median (50th percentile). The whiskers indicate values within 1.5 times the IQR, and the dots are the outliers.

Overall agreement of penetration between the two instruments was very good (rho=0.84, P<0.001). In the 40 L/min flow rate scenario, eight of the nine masks demonstrated median rates of penetration below 9% for both PM_2.5_ and BC (figure 2). The median penetration of BC ranged from 0.2% (IQR: 0.2%–0.3%) to 20.7% (IQR: 19.6%–22.1%) with the lowest value being for the ‘Yimeijian’ mask and the highest being for the ‘Gucheng Professional AntiHaze W&G’ (‘Gucheng’) mask. Values were similar, but slightly higher, using 80 L/min, ranging from 0.7% (IQR: 0.4%–1.0%) to 32.8% (IQR: 21.1%–35.3%) with the same masks performing best/worst.

Penetration of PM_2.5_ was typically greater than for BC: the Yimeijian mask exhibited the lowest median penetration at 0.3% (IQR: 0.2%–0.3%) for the 40 L/min flow, with the highest corresponding penetration seen for the Gucheng, with a median of 29.3% (IQR: 27.7%–30.3%). The median range for 80 L/min tests extended from 0.8% (IQR: 0.7%–1.1%) for Yimeijian to 41.4% (IQR: 33.9%–50.2%) for Gucheng.

### Volunteer study

The 3M9322, 3M9502, Yi Jie PM2.5 mask and the Yimeijian mask were selected for the volunteer trials (figure 1).

**Figure 3:**
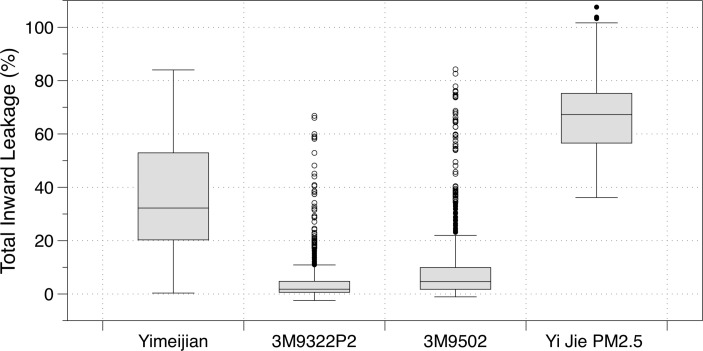
Boxplot showing the total inward leakage for each mask tested in the volunteer trial. Boxes indicate the 25th–75th percentiles (the IQR), and the lines inside the boxes indicate the median (50th percentile). The whiskers indicate values within 1.5 times the IQR, and the dots are the outliers.

There was relatively little variability in face size among volunteers, with the exception of two, which were classified as large (online supplementary table S3). The eight other volunteers were classified as having ‘Small’ or ‘Medium’ faces. Figure 3 provides the TIL ranges for the four masks tested in the volunteer studies, in the same order shown in figure 2. TIL values in excess of 100% are possible where there is a very poor facial seal; in this instance, TIL values would largely represent fluctuations of similar concentration levels inside and outside the mask. A number of high outliers are evident in the TIL values for both 3M masks; the TIL values exceeding 30% occurred for a single subject for each 3M mask, although the volunteers differed between masks. There was clear variation in the unadjusted median TILs across the four masks tested, spanning from 1.8% (IQR: 0.6%–4.7%) for 3M9322 to 67.3% (IQR: 56.6%–75.2%) for the Yi Jie PM2.5 mask.

**Figure 4:**
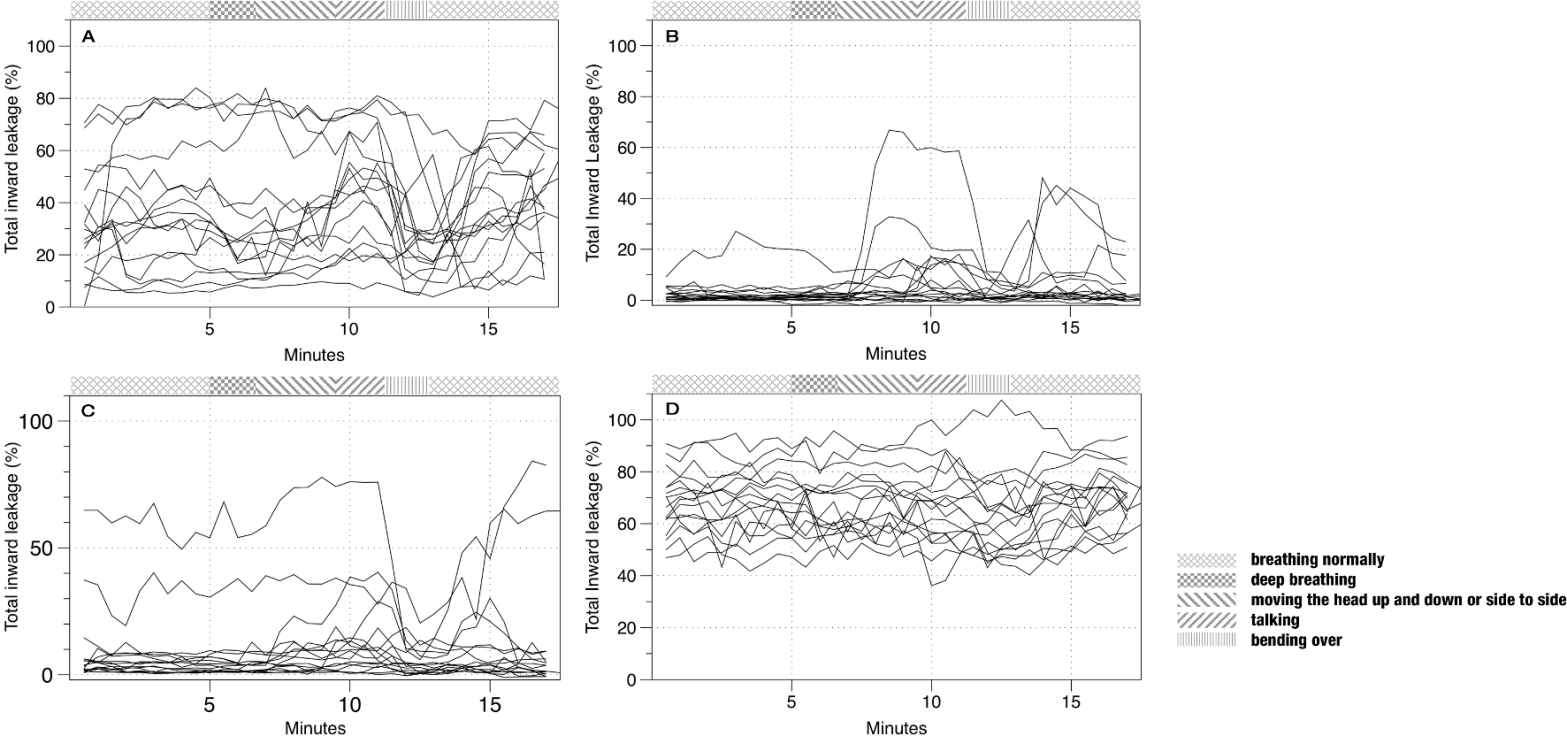
Total inward leakage (TIL) for the four masks tested in the volunteer trial. Each line indicates the TIL over time for one volunteer. A = Yimeijian; B = 3M9322; C = 3M9502; D = Yi Juie PM2.5

There was apparent variation in the TIL across volunteers, time and activities (figure 4). The Durbin-Watson statistic was 0.12 from a simple regression model with mask type as the dependent variable, indicating positive autocorrelation. The transformed value produced in a Cochrane-Orcutt regression was close to 2.00 (1.85), suggesting autocorrelation had been sufficiently addressed. Only mask type and activity were significant in the model at P<0.05. Face size, sitting/standing and elapsed time had no measureable effect on TIL. A log-linear model was deemed a better fit than a linear model according to the Bayesian information criterion difference. After adjustment for covariates, the 3M9322 mask was significantly better than the three other masks, which increased leakage by a factor of 2.0 (95% CI 1.2 to 3.3) to 26.7 (95% CI 16.4 to 43.5) (see table 1). Compared with talking, only bending and head moving contributed to higher TIL, ranging from 1.1 (95% CI 1.0 to 1.3) to 1.2 (95% CI 1.1 to 1.3) times greater than the reference category of talking, respectively. After adjustment for autocorrelation, the regression model explained 11% of the variation in the TIL data (table 1).

**Table 1:** Cochrane-Orcutt log-linear regression analysis results for predictors of total inward leakage (TIL)

Variable	Coefficient for TIL increase	95% CI	P values
Mask			
3M9322	reference	–	–
3M9502	2.0	1.2 to 3.3	0.007
Yimeijian	13.9	8.5 to 22.7	<0.001
Yi Jie PM2.5	26.7	16.4 to 43.5	<0.001
Activity			
Talking	reference	–	–
Breathing deeply	1.0	0.9 to 1.2	0.812
Stationary	1.1	0.9 to 1.2	0.329
Bending	1.1	1.0 to 1.3	0.014
Head moving	1.2	1.1 to 1.3	0.001
Constant n = 2084, R^2^= 0.11	2.3	1.6 to 3.3	<0.001

The coefficients represent the factor by which TIL is increased compared with the reference category.

## Discussion

We assessed the real-life effectiveness of masks worn by Beijing residents, to protect against urban air pollution. We demonstrate that a mask with highly efficient particle filtering material may confer little protection when worn. Facial fit and movement have a large impact on the actual protection. In spite of the generally good filtration efficiency of the tested mask materials, two of the masks performed poorly when worn because of inadequate fit to the face. These included a reusable mask with disposable filters (Yi Jie PM2.5) and a single-fold disposable mask (Yimeijian). The reusable mask cloth was loose, and the filters did not cover the whole surface area of the mask. These masks used elasticated ear loops to hold them onto the face rather than head straps. Even for the better-performing masks (3M9502 and 3M9322), some volunteers’ results indicated that the fit was not sufficient to ensure maximal performance of the mask.

Our first-order autoregression model suggested the presence of large differences in the efficacy of the tested masks, and that activities, namely, bending and head moving, adversely affected the fit. The best performing mask had an estimated mean leakage of 2.3%, which corresponds to a protection factor of 43 (protection factor being the inverse of the leakage, or TIL). The poorest performing mask had a mean leakage of 61% (protection factor 1.6). The face size of the volunteer or the duration did not have a detectable effect on leakage; however, the tests were conducted over a short time, and we cannot be certain of the effect of longer duration wear.

Our results correspond with findings from van der Sande *et al*,^22^ who tested an FFP2 mask (online supplementary table S1) versus a surgical mask and a home-made tea cloth mask against sodium chloride (NaCl) particles <1 µm diameter on volunteers. They found differences between protection factors for volunteers within a single mask type, with children much less protected compared with adults. Variation of protection by activity was greatest for an FFP2 mask compared with surgical and home-made tea cloth masks, implying that, although the FFP2 mask conferred greatest protection in terms of its material composition, fit was an important factor. A study of respiratory protection against volcanic ash also found that industry-certified N95 or N99 equivalent masks performed best in volunteer TIL tests compared with masks made from other materials with similar filtering abilities.^13 14^ Similarly, Grinshpun *et al*^23^ found that 70% of total variability in the faceseal-leakage-to-filter (FLTF) ratio was associated with subject characteristics, compared with 30% due to how the subject put on the respirator. They also found that movement exercises resulted in larger FLTF ratios and that the ratio increased as particle size increased (from 0.04 to 1 µm). Conversely, Lee *et al*^24^ did not find a particle-dependent protection relationship in a similar size range, and Shakya *et al*^25^ found variable results with regard to filtration efficiency and particle size (all below 1 µm). These size-dependent effects may be due to submicron-sized particles being more likely to deposit by diffusion. Diesel exhaust particulate primarily consists of the ultrafine fraction of the aerosol, which can be breathed deep into the alveolar region of the lung and may then cross into the bloodstream.^26^

The use of a diesel exhaust challenge, rather than sodium chloride, provides a scenario that is closer to real-world exposures, particularly near busy roads, where people are more likely to wear masks. Gao *et al*^27^ found that N95 filters did not perform as well compared with R95 or P95 filters when exposed to combustion source aerosol (wood, paper and plastic) compared with NaCl, although all these filters did filter at least 95% of the aerosol.

None of the masks tested in our study were certified to the Chinese guidelines for ‘daily protective masks’ (GB/T 32 610–2016), because the guidelines were introduced after our study was conducted, but six of the nine masks we tested were reportedly certified to GB2626-2006, or an international equivalent. One of these (Yi Jie) performed poorly in the volunteer trial. While respirators for workplace use are required to be tested to a rigorous standard, including TIL, respirators for the consumer market are not.

An analysis of internet purchases of face masks in urban areas across China found that a 100-point increase in the Air Quality Index resulted in a 70% increase in sales of masks marketed as ‘anti-PM_2.5_’ and a 54% increase in all mask purchases,^28^ indicating that this is a popular method of protection used by people in China. To ensure that the general public are able to effectively protect themselves, all consumer air pollution masks should be required to fulfil the same standards as workplace masks, and sales of non-compliant models should be restricted. Consumers in China also need better information about the type of masks that should be used, the actual TIL that might be achieved with face masks and how to wear the masks to achieve a good fit. Consumers should know that, although surgical masks may have reasonable filtration efficiency, the design generally confers poor facial fit and high TIL with regards to air pollution.^25 29 30^

In addition to consumer applications, our study has implications for research studies that investigate health benefits of mask-wearing to protect against air pollution. While studies have shown some reduction in blood pressure and increases in heart rate variability from wearing a respirator over a few hours, there was no assessment of the exposure reduction while the respirators were being worn in any of these studies.^4–6^ Our study has shown that there may be considerable interindividual and intraindividual variation in TIL, even with relatively well-constructed respirators, and there are potentially large, systematic differences among respirator brands.

### Limitations

Our study was not a comprehensive survey of air pollution face masks, but rather, provides preliminary results. We selected a convenience sample of masks from retail outlets based on accessibility and design type. There are a wide variety of masks on the market, and we were not able to test all of them. We also had a relatively small sample size of volunteers, and not all volunteers wore all four masks that were being tested; therefore, we cannot draw conclusions about the fit across all tested masks for a given face shape. We had more small/medium face types than large and did not have sufficient volunteers to distinguish small from medium. We had similar numbers of males and females but, due to small numbers, this was not a representative sample, nor were the subjects exclusively Chinese.

Another potential uncertainty in our study relates to the response of the instruments to the aerosol concentration. If the response is not linear across the range of concentrations, we may be underestimating or overestimating the filtration efficiency. We have previously evaluated the MicroAeth AE51 in the field versus a reference aethelometer (AE22) and found a consistently linear relationship across the range of concentrations tested in this study (unpublished data). Although we do not have a reference comparison for the Sidepak for PM_2.5_, the relationship between the penetration ratios for each instrument is linear, implying that the Sidepak should have a similar response across the range of concentrations in the study.

In future work, we suggest that a comprehensive survey of the different face mask types available and of their popularity of use be conducted. A larger, more representative population sample should be recruited for testing, and a wider variety of face mask types should be tested for TIL. In day-to-day use, respirators will not be constantly worn, and this will inevitably reduce the overall protection offered although, to our knowledge, there are no extensive study data available on wearing behaviour of consumers in China. To assess the exposure-reduction potential of real-world face mask use in the public, it is necessary to understand wearing patterns and to assess the effect of constant donning and doffing of masks by the general public. In future studies of the public health benefits of wearing respiratory protection, we recommend the researchers monitor particulate concentrations inside the facepiece and take these data into account in analysis of the association between exposure to particulate pollution and health markers. This would provide a more accurate assessment of particulate exposure change and more robustness to the evaluation of the exposure–response relationship.
