# Supplementary material for: Effectiveness of face masks used to protect Beijing residents against particulate air pollution
Source: Occup Environ Med. 2018 Apr 9;75(6):446–52. doi: 10.1136/oemed-2017-104765 (PMC5969371; doi:10.1136/oemed-2017-104765)
Supplement: Supplementary file 1 [file oemed-2017-104765supp001.docx]

**Supplemental Information**

**The effectiveness of facemasks used to protect Beijing residents against particulate air pollution**

John W. Cherrie^1,2^, Andrew Apsley^1^, Hilary Cowie^1^, Susanne Steinle^1^, William Mueller^1^, Chun Lin^3^, Claire J. Horwell^4^, Anne Sleeuwenhoek^1^, Miranda Loh^1*^

1. Institute of Occupational Medicine, Centre for Human Exposure Science, Edinburgh, United Kingdom.
2. Heriot Watt University, Institute of Biological Chemistry, Biophysics and Bioengineering, United Kingdom.
3. School of Chemistry, University of Edinburgh, Edinburgh, United Kingdom.
4. Institute of Hazard, Risk & Resilience, Department of Earth Sciences, Durham University, Durham, United Kingdom.

[Table S1: Respiratory protection legislation for particulate filtering facepieces. Standards are compulsory, guidelines are recommendations, not compulsory. 3](#_Toc500946416)

[Table S2: Volunteer facial measurements 4](#_Toc500946417)

[Figure S1: Schematic diagram of the filtration efficiency test apparatus. Internal dimensions of the chamber were 0.50 m (L) x 0.25 m (W) x 0.12 m (H). 7](#_Toc503363658)

[Figure S2: Schematic diagram of the apparatus for the volunteer tests to assess TIL. Dimensions of the chamber were 2.5m x 2.5m x 2.4m. 8](#_Toc503363659)

[Figure S3: Sampling probe attached to mask from outside (A) and inside (B) 10](#_Toc503363660)

[Figure S4: Mask, sampling probe, and headgear set up on mannequin 11](#_Toc503363661)

Table S1: Respiratory protection legislation for particulate filtering facepieces. Standards are compulsory, guidelines are recommendations, not compulsory.

| Standard or Guideline | Geographical legislation | Protection classes | Summary |
| --- | --- | --- | --- |
| EN149:2001^a^ (standard) | Europe | FFP1  FFP2  FFP3 | For solid, liquid, oil based aerosols. Protection level that should be achieved if the respirator is a good fit and is used correctly. Should reduce the amount of aerosol inhaled by factors of at least 80% (FFP1), 94% (FFP2) and 99% (FFP3). TIL test^b^ required. |
| 42 CFR 84^c^ (standard) | United States | N95, 99, 100 (Not resistant to oil)  R95, 99, 100 (Resistant to oil)  P95, 99, 100 (Oil Proof) | Filtration efficiency of 95, 99, 99.97% against particulate matter of varying types. No TIL test required. |
| GB2626-2006^d^ (standard) | China | KN90, 95, 100 | Filtration efficiency of 95, 99, 99.97% against particulate matter, not resistant to oil (KP protects against oily particulate). TIL test required. |
| GB/T 32610-2016^d^ (guideline) | China | A: 90% (≤ 500 µg/m^3^)^e^  B: 85% (≤ 350 µg/m^3^) ^e^  C: 75% (≤ 250 µg/m^3^) ^e^  D: 65% (≤ 150 µg/m^3^)^e^ | Daily protective masks for general use against PM2.5. Classes based on filtration efficiency test on different sized mannequin heads. No TIL test required. |

^a^European standards (EN)

^b^Total Inward Leakage test panel required to be passed.

^c^Code of Federal Regulations

^d^Indicates “Guobiao” for standards issued by the Standardization Administration of China

^e^Concentrations indicate highest levels up to which the masks of specified classes are recommended to be used.

Table S2: Masks tested in this study along with information about mask features as noted by the mask packaging. Standard and protection level descriptions in Table S1.

| ID | Mask Name | Integrated Exhalation Valve | Material | Strap type | Listed Standard related to device performance | Protection level on packaging | Hazards protected against^a^ | Volunteer Study |
| --- | --- | --- | --- | --- | --- | --- | --- | --- |
| A | 3M8210 | Yes | Non-woven^b^ | Over head straps | GB2626-2006^c^  NIOSH^d^ 42 CFR^e^ 84 | KN95^f^ | Particulate |  |
| B | 3M9001 | Yes | Non-woven^b^ | Ear straps | GB2626-2006^c^ | KN90^g^ | PM2.5 |  |
| C | 3M9322 | Yes | Non-woven^b^ | Over head straps | EN149-2001^h^  AS/NZ 1716:2012 P2^i^ | FFP2 NR D^j^ | Particulate | √ |
| D | 3M9501 | Yes | Non-woven^b^ | Ear straps | GB2626-2006^c^ | KN95^e^ | Particulate |  |
| E | 3M9502 | Yes | Non-woven^b^ | Over head straps | GB2626-2006^c^ | KN95^e^ | Particulate | √ |
| F | Green Shield G&W | No | Cotton with removable filter | Ear straps | None | None | PM2.5 |  |
| G | Yi Jie PM2.5 | No | Cotton with removable filter | Ear straps | GB2626-2006^c^ | >99% | PM_>0.3_, vehicle exhaust, indirect smoking, benzene, bacterial, virus, etc. | √ |
| H | Gucheng Professional AntiHaze W&G | No | Cotton with removable filter | Ear straps | None | None | PM2.5 Antibiotic UV Protection Anti Haze |  |
| I | Yimeijian | Yes | Non-woven^b^ | Ear straps | None | None | PM, haze, dust, droplets, odour | √ |

^a^As stated on the packaging or according to the standard

^b^ “A manufactured sheet, web or batt of directionally or randomly orientated fibres, bonded by friction, and/or cohesion and/or adhesion…” (ISO, 2011)

^c^Indicates “Guobiao” for standards issued by the Standardization Administration of China

^d^National Institute for Occupational Safety and Health of the USA

^e^Code of Federal Regulations

^f^KN95: Designation of filtration efficiency less of 95% for non-oily particulate for GB2626-2006 standard

^g^KN90: Designation of filtration efficiency less of 90% for non-oily particulate for GB2626-2006 standard

^h^European standards (EN)

^i^Australian and New Zealand standards

^j^European standard designation that respirator should reduce amount of aerosol by at least 94%, if respirator is a good fit and worn correctly

Table S3: Volunteer facial measurements

| **Subject** | **Sex** | **Age** | **Lip Length (mm)** | **Menton Sellion length (mm)** | **Bizygomatic Width (mm)** | **Tragion to the tip of the nose (mm)** | **Size Classification** |
| --- | --- | --- | --- | --- | --- | --- | --- |
| **1** | Female | 19 | 49 | 104 | 139 | 109 | Small |
| **2** | Female | 42 | 51 | 109 | 142 | 113 | Medium |
| **3** | Male | 20 | 49 | 112 | 141 | 120 | Medium |
| **4** | Male | 20 | 54 | 130 | 160 | 132 | Large |
| **5** | Male | 20 | 53 | 111 | 143 | 123 | Medium |
| **6** | Male | 62 | 53 | 135 | 147 | 140 | Large |
| **7** | Female | 29 | 49 | 106 | 143 | 111 | Small |
| **8** | Female | 31 | 49 | 111 | 154 | 111 | Medium |
| **9** | Female | 37 | 51 | 115 | 136 | 111 | Medium |
| **10** | Female | 40 | 43 | 115 | 136 | 114 | Medium |

**
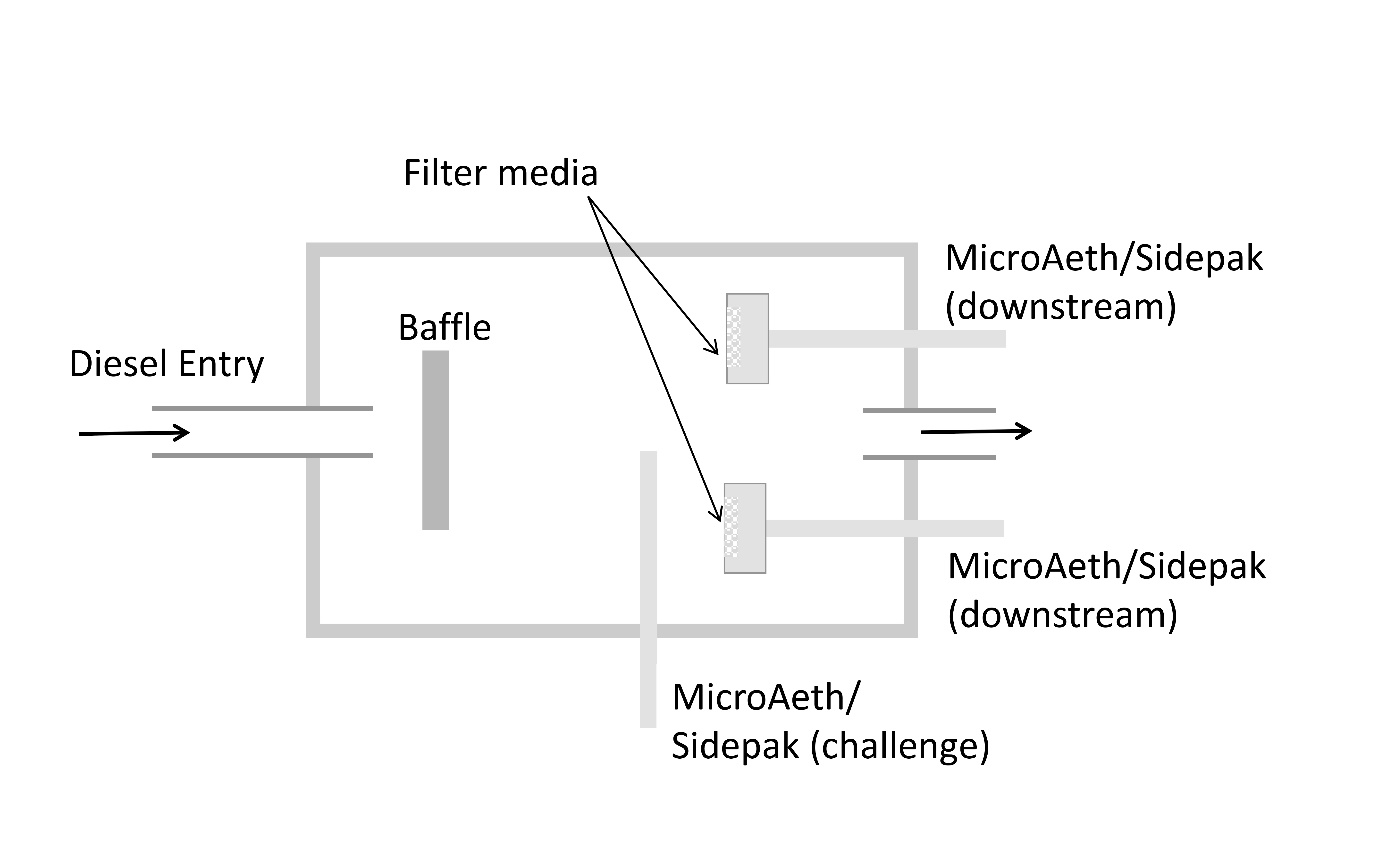
**

Figure S1: Schematic diagram of the filtration efficiency test apparatus. Internal dimensions of the chamber were 0.50 m (L) x 0.25 m (W) x 0.12 m (H).

Each sample holder was connected to a MicroAeth AE51 (Aethlabs, San Francisco, USA), running at 200 mL/ min, to measure black carbon, and a Sidepak AM510 (TSI, Minnesota, USA) running at 1.7 L/min to measure PM_2.5_ (Figure S2). The Sidepak was not calibrated for diesel exhaust because we were examining the relative change in filtration, rather than absolute concentrations. A MicroAeth (50 mL/min) and Sidepak (1.7 L/min) measured the chamber challenge concentrationevery 30 seconds. A pump made up the required overall flow (Casella CEL, Bedford, UK, pump for 40 L/min; SKC MCS FLITE model 901-20, Pennsylvania, USA pump for 80 L/min). Flowrates were tested and adjusted with a TSI 4100 series flowmeter.


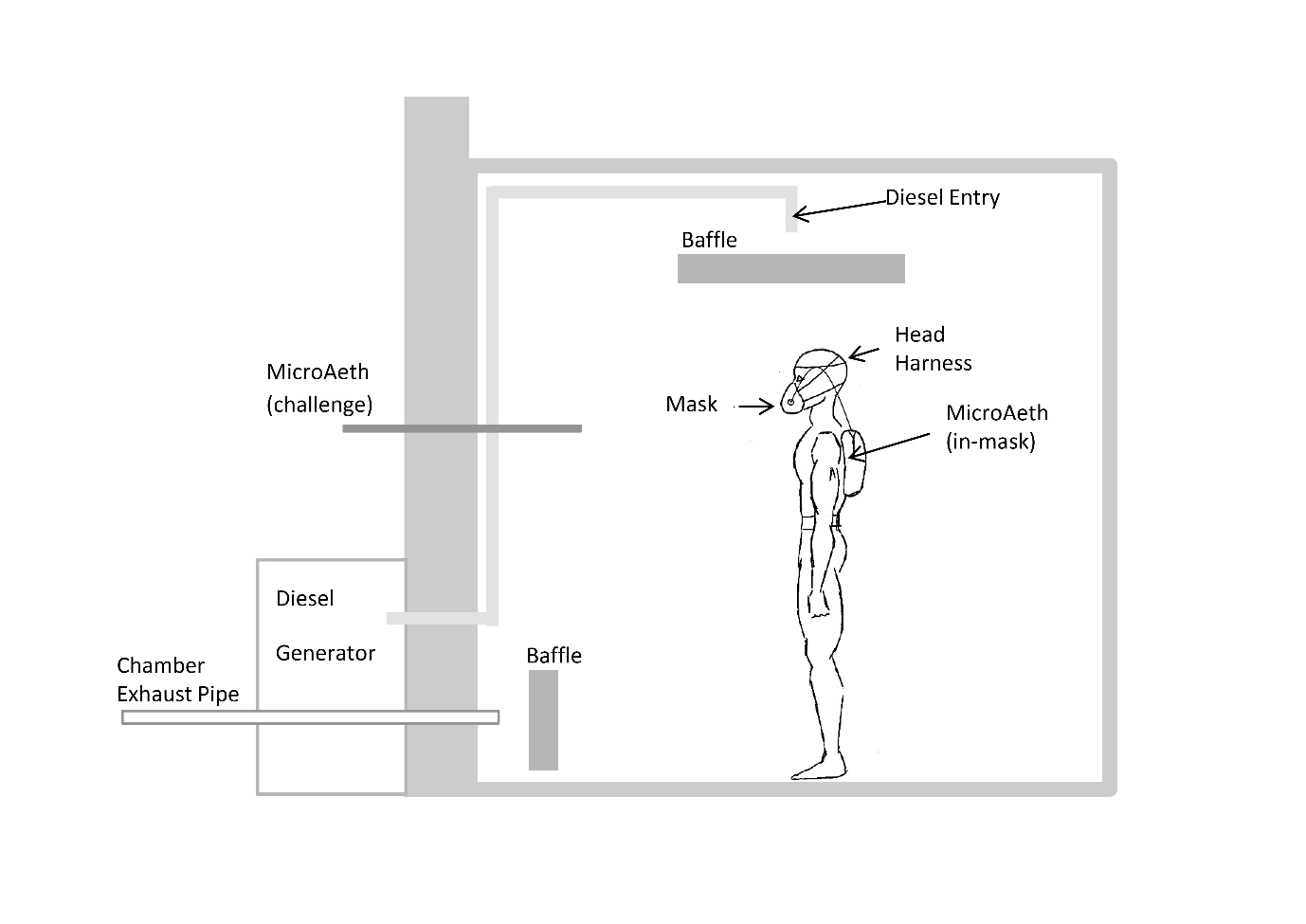


Figure S2: Schematic diagram of the apparatus for the volunteer tests to assess TIL. Dimensions of the chamber were 2.5m x 2.5m x 2.4m.

Volunteers were provided with the mask, and access to the instructions (which included pictorial instructions) on the packaging. The volunteers were asked to put on the mask and photos were taken from different angles of the volunteer wearing the mask. Then, the mask was removed and the sampling probe inserted through the mask material and secured to prevent leakage around the hole (Figure S3). The probe was attached to a tube connected to a headgear, to help reduce any distortion effects of the tube and probe on the mask. The tube was connected to the MicroAeth, which was placed in a small backpack. Then the volunteer was allowed to put the mask on by themselves, although the researcher helped them with the process of getting the headgear and backpack on. Finally, the researcher ensured that the probe, tubing, and headgear were correctly and securely positioned. The sampling probe was positioned around the nose and mouth (Figure S4). The probe was a 20 mm aluminium disc with eight equidistant 1 mm inlet points on the outside of the disc to prevent impaction of the inlet points with the face according to BS EN 140:1999 [17] (Figure S4). Researchers noted down contextual information about anything that may have affected the protection afforded by the mask, although this was not used in data analysis.


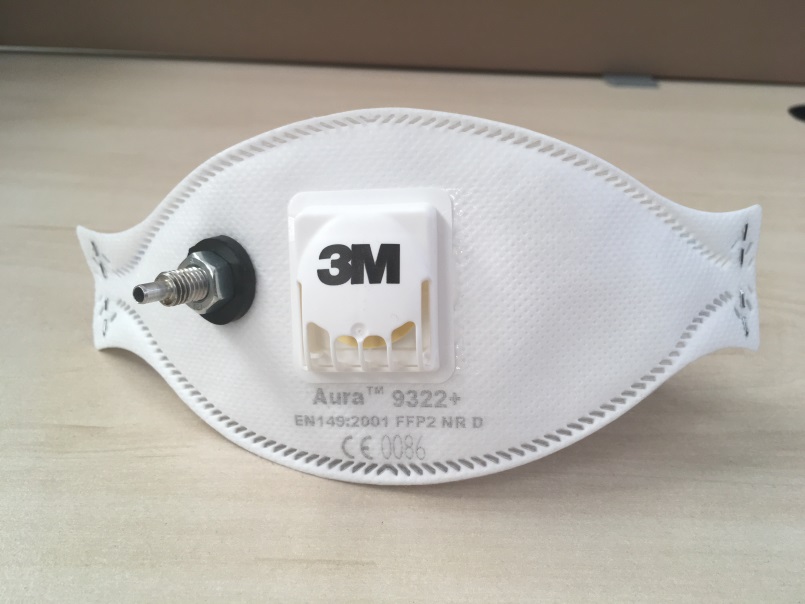


A


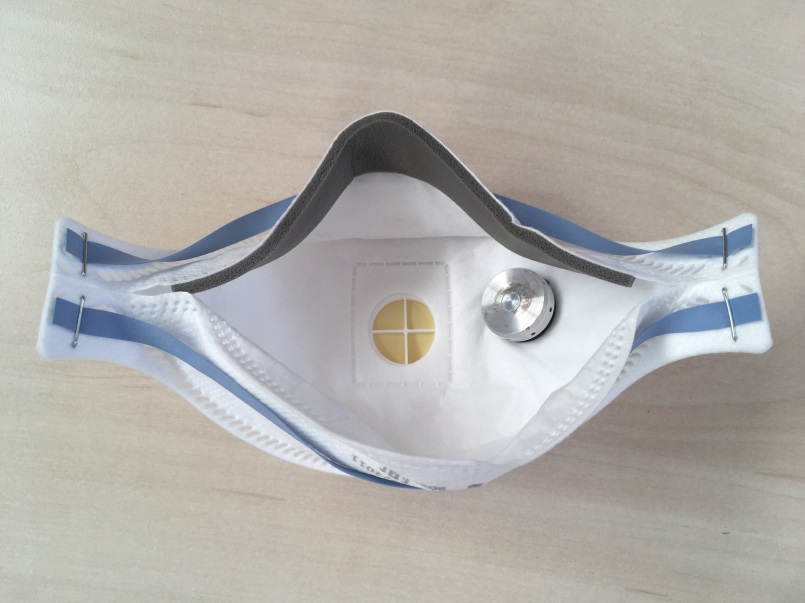


B

Figure S3: Sampling probe attached to mask from outside (A) and inside (B)


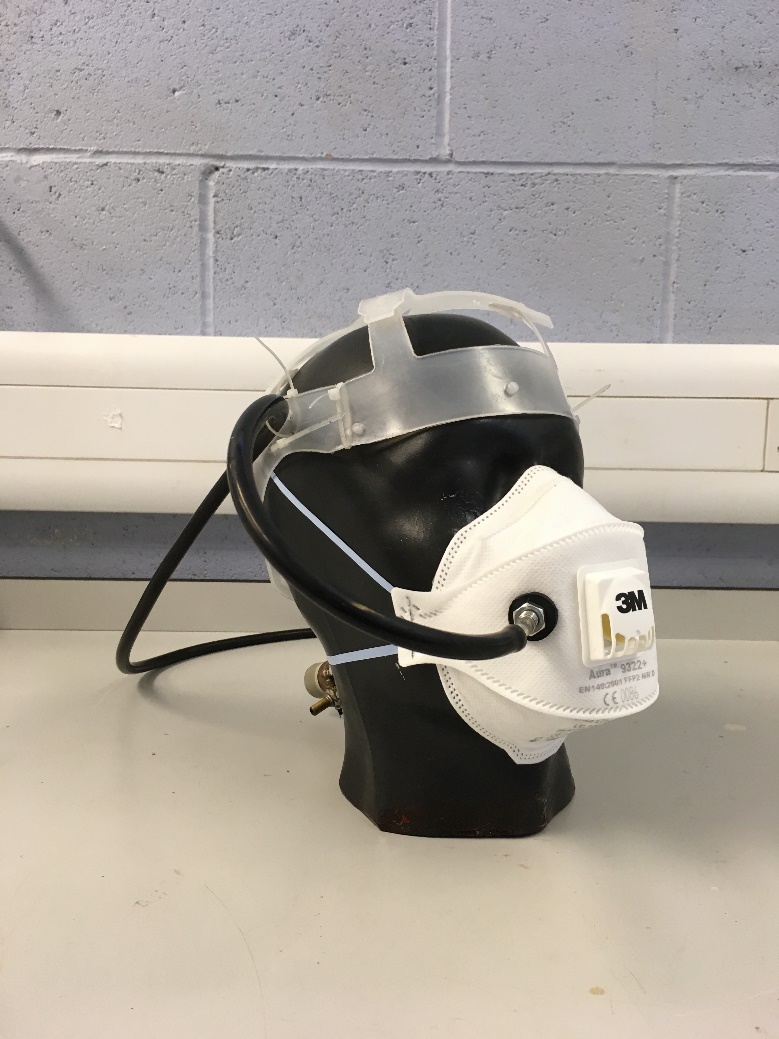


Figure S4: Mask, sampling probe, and headgear set up on mannequin
